# Inflammatory biomarker-based prognostic model for immunotherapy outcomes in patients with recurrent or metastatic cervical cancer

**DOI:** 10.3389/fonc.2026.1818240

**Published:** 2026-06-12

**Authors:** Yulu Wang, Chengyang Luo, Yueze Li, Jiayu Pei, Dian Wang, Pei Wang, Jing Li

**Affiliations:** 1 Department of Oncology, the First Affiliated Hospital of Zhengzhou University, Zhengzhou, Henan, China; 2 Department of Gynecology, the First Affiliated Hospital of Zhengzhou University, Zhengzhou, Henan, China

**Keywords:** cox regression, least absolute shrinkage and selection operator, monocyte-to-lymphocyte ratio, neutrophil-to-lymphocyte ratio, platelet-to-lymphocyte, programmed death-1/programmed death ligand-1 inhibitor, recurrent or metastatic cervical cancer, systemic immune-inflammation index

## Abstract

**Background:**

Conventional predictive biomarkers, including programmed death ligand-1 (PD-L1) expression and tumor mutational burden testing, fail to predict immunotherapy efficacy in patients with recurrent or metastatic cervical cancer (r/m CC), highlighting the need for accessible and dynamically evaluable indicators.

**Objective:**

This study aimed to develop an inflammatory biomarker-based prediction model to identify patients with advanced CC who may benefit from anti-PD-1/PD-L1 therapy.

**Methods:**

A total of 263 patients with r/m CC receiving immunotherapy were randomly divided into training (70%, n = 185) and validation (30%, n = 78) cohorts. The maximum log-rank test was used to determine optimal cutoff values for pretreatment inflammatory markers (neutrophil-to-lymphocyte ratio, platelet-to-lymphocyte ratio, monocyte-to-lymphocyte ratio, and systemic immune-inflammation index); posttreatment markers were stratified by comparison with baseline levels. Least absolute shrinkage and selection operator (LASSO) regression was applied for feature selection, and the selected variables were incorporated into a Cox proportional hazards model to establish the prognostic model. Model performance was evaluated using time-dependent receiver operating characteristic (ROC) curves, concordance indices, calibration curves, and decision curve analysis.

**Results:**

Optimal cutoff values for pretreatment neutrophil-to-lymphocyte ratio, platelet-to-lymphocyte ratio, monocyte-to-lymphocyte ratio, and systemic immune-inflammation index were 2.82, 243.41, 0.39, and 580.10, respectively. The LASSO-Cox model showed strong predictive performance. Calibration curves confirmed consistency between predicted and observed overall survival (OS) or progression-free survival (PFS). Time-dependent ROC curves yielded training set area under the ROC curve values (1-, 2-, and 3-year) of 0.700, 0.740, and 0.759 (OS) and 0.732, 0.835, and 0.757 (PFS), respectively, as well as validation set area under the ROC curve values of 0.715, 0.771, and 0.803 (OS) and 0.732, 0.835, and 0.757 (PFS), respectively. Decision curve analysis demonstrated stable net clinical benefit. A nomogram was developed, with Kaplan–Meier analysis showing poorer OS or PFS in high-risk patients stratified by the nomogram.

**Conclusion:**

The inflammatory biomarker-based prognostic model predicts immunotherapy outcomes in patients with r/m CC, providing reliable clinical guidance for treatment decision-making.

## Introduction

Cervical cancer (CC) is one of the most common malignant tumors in women, imposing a substantial disease burden. In 2022, approximately 660,000 new cases of CC were diagnosed worldwide, with 350,000 associated deaths. China reported 150,700 new cases and 55,700 deaths from CC in 2022, accounting for ~16% of the global burden (1). In recent years, the popularization of high-risk human papillomavirus (HPV) screening programs and the expanded coverage of HPV vaccines have directly improved the effectiveness of early diagnosis and treatment and indirectly increased overall prognosis in patients with CC (1, 2). However, the 5-year survival rate of patients with recurrent or metastatic CC (r/m CC) is only 17%, presenting significant challenges to clinical treatment (3).

In the clinical management of r/m CC, patients exhibit diverse clinical manifestations, including isolated recurrence, isolated metastasis, or extensive metastasis combined with recurrence. Due to the high risk of further metastasis in the patients, systemic drug therapy has become the core treatment strategy recommended in clinical practice (3). In 2024, the GOG169 study identified cisplatin combined with paclitaxel as the standard chemotherapy regimen for r/m CC (4). Advances in research on the pathogenesis and progression of CC have identified persistent infection with high-risk HPV subtypes as a key driver of most CC cases. Integration of the E6 and E7 HPV oncoproteins into the host cell genome dysregulates their expression, creating favorable conditions for subsequent mutations that induce tumor cell survival and immune escape (5). This state of immune system suppression serves as a key link in CC progression. Extensive expression of programmed death ligand-1 (PD-L1) in the tumor microenvironment directly inhibits cellular immune function, thereby facilitating immune escape and accelerated proliferation of tumor cells (6, 7). Additionally, the presence of tumor-infiltrating immune cells exerts a significant effect on the prognosis of patients with CC (8). The interaction between HPV infection and the host immune system has attracted extensive attention to the application of immunotherapeutic agents in CC treatment.

In June 2018, pembrolizumab became the first immunotherapeutic agent to receive accelerated approval from the U.S. Food and Drug Administration as a second-line treatment for PD-L1-positive persistent or r/m CC (9). In October 2021, the U.S. Food and Drug Administration further approved pembrolizumab as a first-line treatment for PD-L1-positive r/m CC (10). Since then, the regimen of pembrolizumab combined with chemotherapy and bevacizumab has become the standard first-line treatment for patients with PD-L1-positive r/m CC (combined positive score ≥ 1), confirming that PD-L1 can serve as an important biomarker for predicting pembrolizumab efficacy in patients with CC. However, studies have revealed significant inconsistencies in PD-L1 expression between sites of primary tumors and those of recurrent or metastatic disease. Liu et al. reported that the PD-L1-positive rate was significantly lower in primary lesions (15.4%) than in recurrent or metastatic lesions (30.4%) (11). Another study showed that the objective response rate of patients with PD-L1-negative tumors reached 17.9%—nearly equivalent to the 16.7% observed in patients with PD-L1-positive tumors—indicating that PD-L1 negativity is not an absolute contraindication for immunotherapy (12). As easily accessible assessment indicators, peripheral blood inflammatory markers can reflect the balance between the host’s inflammatory response and immune surveillance, thereby illustrating the interactions between inflammation, immune function, and tumors (13). Several studies have verified that peripheral blood indicators, such as neutrophil-to-lymphocyte ratio (NLR), monocyte-to-lymphocyte ratio (MLR), and platelet-to-lymphocyte ratio (PLR), have definite predictive value for immunotherapy efficacy in malignancies (14–19).

This study retrospectively analyzed the clinical data and peripheral blood indicators of 263 patients with advanced CC who received anti-PD-1 or anti-PD-L1 therapy. The objectives of this study were to identify patients with advanced CC who could benefit from anti-PD-1/PD-L1 therapy and to confirm effective predictive biomarkers for treatment efficacy.

## Methods

### Patients

This study enrolled 263 patients with r/m CC who received immunotherapy at the First Affiliated Hospital of Zhengzhou University between September 2018 and July 2024, based on the following inclusion and exclusion criteria. The inclusion criteria are as follows: 1) histologically confirmed squamous cell carcinoma, adenocarcinoma, or adenosquamous cell carcinoma; 2) received anti-PD-1 therapy with or without other concurrent treatments (anti-PD-1 agents including nivolumab, pembrolizumab, sintilimab, camrelizumab, tislelizumab, and toripalimab); 3) serological data available before immunotherapy initiation and at the third treatment cycle; and 4) complete baseline clinical data available. The exclusion criteria are as follows: 1) intolerance to immunotherapy; 2) administration of drugs targeting hematopoietic function within 2 weeks before the first immunotherapy cycle and 2 weeks before the third treatment cycle; and 3) incomplete clinical data. This study was conducted in strict compliance with the ethical principles stipulated in the Declaration of Helsinki. Written informed consent was obtained from each patient or their legal representatives before collecting and using clinical data for statistical analysis. All data were anonymized, and the study was approved by the Institutional Ethics Review Board of the First Affiliated Hospital of Zhengzhou University (Approval No. 2026-KY-0044).

### Serological detection markers

Serum samples were collected from patients within 1 week before the first cycle of immunotherapy and within 1 week before the third cycle of immunotherapy. The definitions of inflammatory ratios are as follows: NLR is the ratio of peripheral blood neutrophil count to lymphocyte count; PLR is the ratio of peripheral blood platelet count to lymphocyte count; MLR is the ratio of peripheral blood monocyte count to lymphocyte count; and systemic immune-inflammation index (SII) is the ratio of the product of peripheral blood platelet count and neutrophil count to lymphocyte count.

### Follow-up

Follow-up was performed by reviewing patients’ outpatient and inpatient re-examination records, combined with telephone follow-up. The follow-up contents included plasma tumor marker tests, imaging examinations (ultrasound, computed tomography, magnetic resonance imaging, and positron emission tomography-computed tomography), as well as the time of disease recurrence and death events. Overall survival (OS) was set as the primary endpoint, defined as the interval from the initiation of anti-PD-1/PD-L1 therapy to the occurrence of death or the date of follow-up cutoff. Progression-free survival (PFS) was the secondary endpoint, defined as the interval from the initiation of anti-PD-1/PD-L1 therapy to disease progression or the date of follow-up cutoff. The deadline for follow-up was July 25, 2024.

### Statistical analysis

All statistical analyses and figure generation were performed using R software (version 4.3.3) and SPSS software (version 26). The chi-square test was applied to compare categorical variables between two groups, presented as frequency (percentage). The independent-samples t-test was used for comparing normally distributed continuous variables, expressed as mean ± standard deviation. The rank-sum test was employed for non-normally distributed continuous variables between two groups, reported as median (range). The optimal cutoff values were determined using the maximum log-rank test. Patients were randomly assigned to a training cohort (70%) and a validation cohort (30%) using a computer-generated fixed-ratio sampling method. Categorical variables between the training and validation cohorts were compared via the chi-square test. The least absolute shrinkage and selection operator (LASSO) regression was conducted in the training cohort for feature selection and predictive model construction. Variables corresponding to the minimum mean squared deviation (MSD) in LASSO analysis were selected as optimal features. Subsequently, Cox proportional hazards regression analysis was performed to establish a Cox regression model for prognostic factors of survival after immunotherapy, and a nomogram was constructed. The concordance index (C-index) for predicting PFS and OS in both the training and validation cohorts were calculated using the “survival” package in R software to evaluate the discriminative ability of the model in ranking patient survival. Time-dependent ROC curves were used to assess the predictive performance of the model for PFS and OS at 1-year, 2-year, and 3-year time points, with corresponding AUC values calculated. Calibration curves were applied to verify the consistency between the predicted risk and actual survival risk via 1000 bootstrap resamplings. Decision curve analysis (DCA) was performed to evaluate the clinical net benefit and practical value of the model at various threshold probabilities, while survival curves were plotted and survival rates estimated using the Kaplan–Meier method, differences in survival were compared using the log-rank test, the proportional hazards assumption of the Cox regression was examined using the Schoenfeld residual test, and if violated, stratified Cox regression was used for adjustment, with a value of P < 0.05 considered statistically significant.

## Results

### Baseline characteristics of patients with r/m CC

**Table 1** presents the baseline clinical characteristics of 263 patients with r/m CC. The patients were randomly assigned to training (n = 185) and validation (n = 78) cohorts at a 7:3 ratio. The training and validation cohorts were used for model development and validation, respectively. No statistically significant differences (*P* > 0.05) were observed between the two cohorts in variables including age, pathological type, pretreatment PD-L1 expression, prior treatment regimens, lines of immunotherapy, and immunotherapy combination strategies. This confirms the randomness and appropriateness of data grouping.

**Table 1:** Clinical baseline characteristics of patients in the training and validation sets.

Characteristic	Patients (n = 263)	Training set (n = 185)	Test set (n = 78)	P-value
Age (years)				
Median	53	52	54	0.184
Range	22–79	22–75	30–78	
Pathology, n (%)				
Squamous cell carcinoma	234 (88.97)	162 (87.57)	72 (92.31)	0.606
No squamous cell carcinoma	29 (11.03)	23 (12.43)	6 (7.69)	
PD-L1 status, n (%)				
Positive	71 (27.00)	49 (26.49)	22 (28.21)	0.748
Negative	32 (12.17)	21 (11.35)	11 (14.10)	
Unknown	160 (60.84)	115 (62.16)	45 (57.69)	
Immunotherapy line, n (%)				
First line^1^	206 (78.33)	146 (78.92)	60 (76.92)	0.845
Second line^2^	57 (21.67)	39 (21.08)	18 (23.08)	
Previous treatment, n (%)				
S3	24 (9.13)	20 (10.81)	4 (5.13)	0.177
S & C^4^	129 (49.05)	85 (45.95)	44 (56.41)	
C^5^	110 (41.83)	80 (43.24)	30 (38.46)	
Combination treatment, n (%)				
Yes	236 (89.73)	163 (88.11)	73 (93.59)	0.368
No	27 (10.27)	22 (11.89)	5 (6.41)	
Pre-NLR, n (%)				
Low (≤2.82)	81 (30.80)	58 (31.35)	23 (29.49)	0.878
High (>2.82)	182 (69.20)	127 (68.65)	55 (70.51)	
Pre-PLR, n (%)				
Low (≤243.41)	136 (51.71)	101 (54.59)	35 (44.87)	0.192
High (>243.41)	127 (48.29)	84 (45.41)	43 (55.13)	
Pre-MLR, n (%)				
Low (≤0.39)	143 (54.37)	108 (58.38)	35 (44.87)	0.061
High (>0.39)	120 (45.63)	77 (41.62)	43 (55.13)	
Pre-SII, n (%)				
Low (≤580.10)	81 (30.80)	59 (31.89)	22 (28.21)	0.656
High (>580.10)	182 (69.20)	126 (68.11)	56 (71.79)	
Post-NLR, n (%)				
Decrease	137 (52.09)	96 (51.89)	41 (52.56)	1.000
Increase	126 (47.91)	89 (48.11)	37 (47.44)	
Post-PLR, n (%)				
Decrease	128 (48.67)	90 (48.65)	38 (48.72)	1.000
Increase	135 (51.33)	95 (51.35)	40 (51.28)	
Post-MLR, n (%)				
Decrease	104 (39.54)	72 (38.92)	32 (41.03)	0.856
Increase	159 (60.46)	113 (61.08)	46 (58.97)	
Post-SII, n (%)				
Decrease	159 (60.46)	114 (61.62)	45 (57.69)	0.648
Increase	104 (39.54)	71 (38.38)	33 (42.31)	

^1^Patients who received immunotherapy after first recurrence or metastasis.

^2^Patients who received immunotherapy after second recurrence or metastasis.

^3^Surgery.

^4^Surgery combined with chemoradiotherapy.

^5^Chemoradiotherapy.

### Optimal cutoff values for NLR, PLR, MLR, and SII

**Figure 1:**
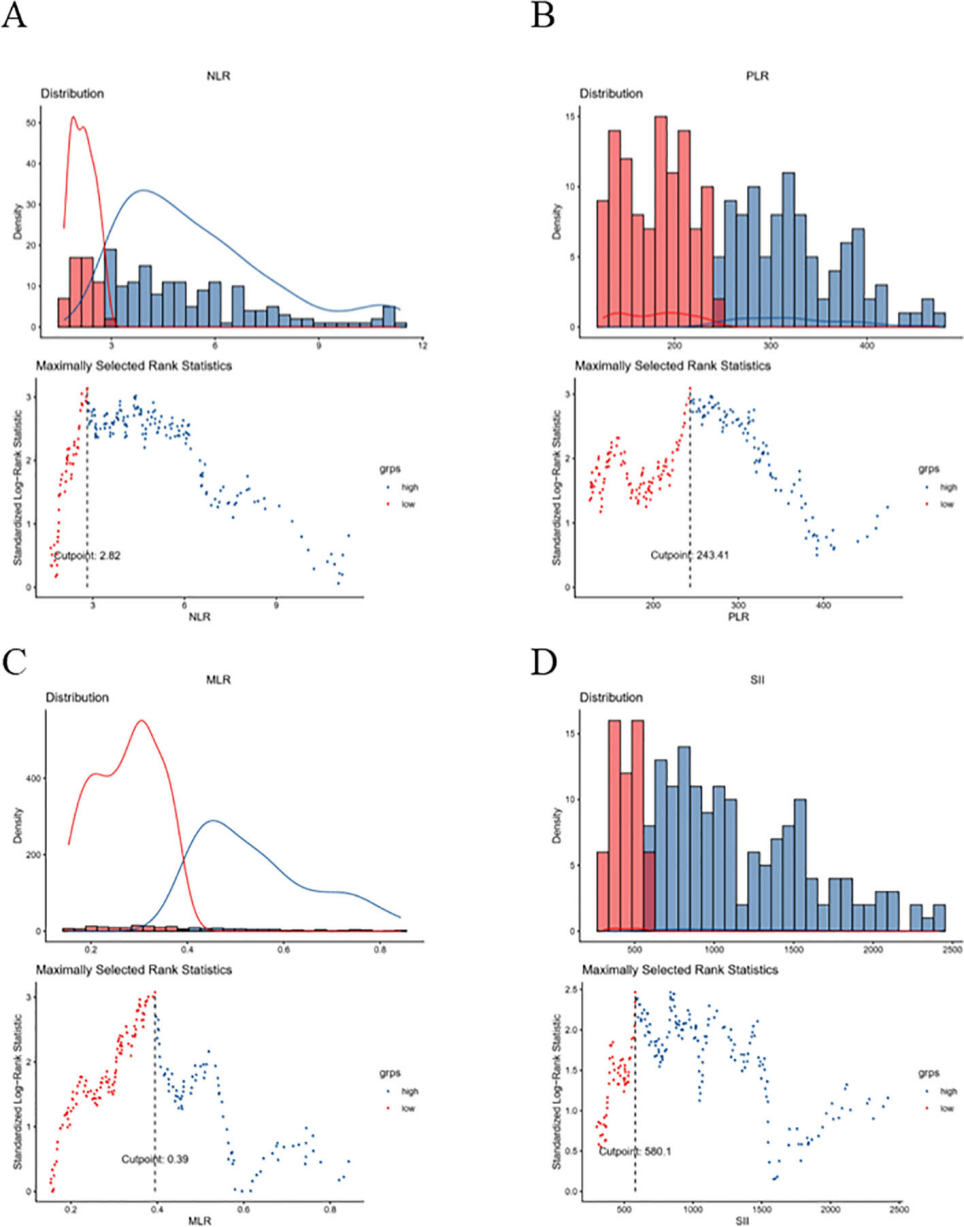
Optimal cutoff values were determined through the maximum log-rank test. **(A)** Optimal cutoff value of pretreatment neutrophil-to-lymphocyte ratio = 2.82. **(B)** Optimal cutoff value of pretreatment platelet-to-lymphocyte ratio = 243.41. **(C)** Optimal cutoff value of pretreatment monocyte-to-lymphocyte ratio = 0.39. **(D)** Optimal cutoff value of pretreatment systemic immune-inflammation index = 580.1.

The maximum log-rank test was used to determine optimal cutoff values for pretreatment inflammatory biomarkers (pre-NLR, pre-PLR, pre-MLR, and pre-SII) in predicting survival outcomes, with thresholds identified as 2.82 (pre-NLR), 243.41 (pre-PLR), 0.39 (pre-MLR), and 580.10 (pre-SII) (**Figure 1**). Based on these cutoff points, each pretreatment biomarker was categorized as low or high (pre-NLR, pre-PLR, pre-MLR, and pre-SII). For posttreatment evaluations, biomarker levels were compared with pretreatment baselines, and each marker was classified as decreased or increased (post-NLR, post-PLR, post-MLR, and post-SII).

### Construction of the predictive model

**Figure 2:**
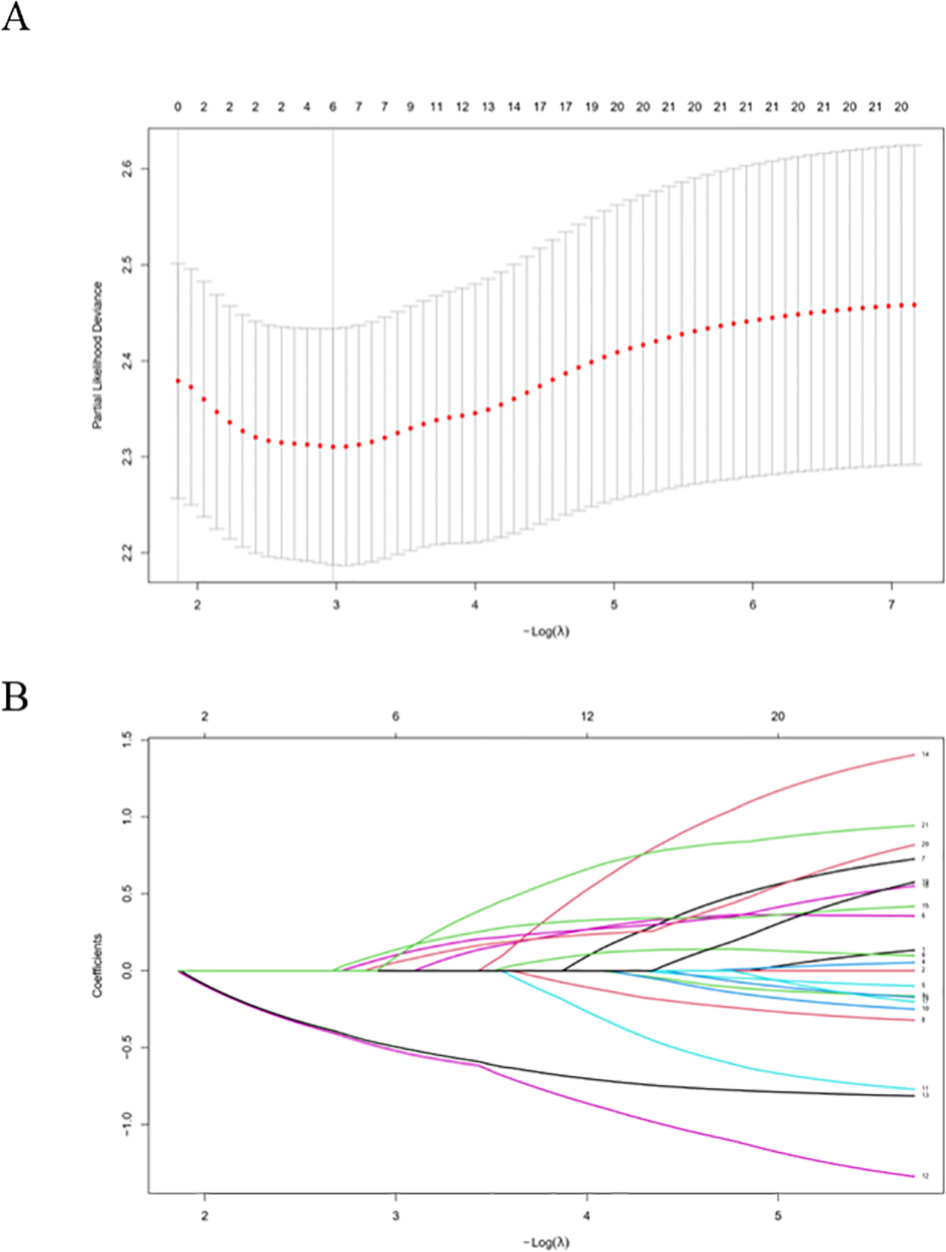
Factor selection using the least absolute shrinkage and selection operator regression. **(A)** Least absolute shrinkage and selection operator coefficient distribution of 21 factors. **(B)** Selection of six prognostic factors by the minimum standard error criterion of optimal lambda.

LASSO regression was applied for feature selection and parameter optimization to develop a predictive model for survival outcome. Binomial deviance curves were plotted with the logarithm of the tuning hyperparameter λ (log(λ)) on the x-axis: the solid vertical line denotes binomial deviance ± standard error, and the dashed vertical lines indicate optimal values derived from the minimum criterion and one standard error criterion, respectively. A 10-fold cross-validation combined with the minimum criterion was used to confirm the optimal hyperparameter for the LASSO model (**Figure 2A**), with the final optimal λ determined as 0.050997 (log(λ) = −2.97). Based on the optimal λ, six variables were selected as core prognostic factors, namely pre-MLR, pre-PLR, post-PLR, post-NLR, age and previous treatment. A coefficient distribution plot was generated across the log(λ) sequence (**Figure 2B**), which illustrates the shrinkage trend of each variable’s coefficient as λ changes and further validates the rationality of the feature selection results. Then, Cox regression analysis was conducted using LASSO-screened variables to construct the prediction model for OS and PFS (**Tables 2** and **3**). The Omnibus test indicated statistical significance for both models (P < 0.001), demonstrating that the incorporated variables had robust combined predictive power for patient prognosis. In the OS model, Pre-PLR (HR = 2.570, 95% CI: 1.494–4.423, P = 0.001) and Pre-MLR (HR = 2.503, 95% CI: 1.485–4.217, P = 0.001) were identified as independent adverse prognostic factors. Surgery combined with chemoradiotherapy trended toward lower event risks (HR = 0.244, 95%CI: 0.590–1.014, P = 0.052) without statistical significance. Other variables, including post-treatment inflammatory index changes, age, and standalone Chemoradiotherapy, showed no independent prognostic value (P > 0.05). For the PFS model, Pre-PLR (HR = 1.789, 95%CI: 1.216–2.631, P = 0.003) and Pre-MLR (HR = 1.986, 95%CI: 1.986, 95%CI: 1.353–2.914, P = 0.001) independently increased progression risk, whereas combined Surgery and chemoradiotherapy served as an independent protective factor (HR = 0.399, 95%CI: 0.171–0.926, P = 0.033). The remaining variables showed no significant independent predictive capability (P > 0.05) but were retained considering their clinical plausibility and contribution to model optimization.

**Table 2:** Cox regression analysis of Lasso-screened prognostic factors.

Variables	B	*P*	*HR* (95%CI)
Pre-PLR (Low/ High)	0.944	0.001	2.570(1.494-4.423)
Pre-MLR (Low/ High)	0.917	0.001	2.503(1.485-4.217)
Post-PLR (Decrease/Rise)	0.485	0.057	0.625(0.985-2.680)
Post-NLR (Decrease/Rise)	0.129	0.607	1.138(0.697-1.858)
Age	0.013	0.238	1.013(0.991-1.036)
Previous treatment (S^*^/ S & C^*^/ C^*^)			
S & C	1.488	0.040	4.430(1.068-18.368)
C	1.395	0.051	4.130(0.995-17.147)

The overall Omnibus test of the model was significant (P < 0.001). Previous treatment with simple surgery was set as the reference group. S*: Surgery; S & C*: Surgery combined with chemoradiotherapy; C*: Chemoradiotherapy.

**Table 3:** Cox Regression Analysis of LASSO-Screened PFS Prognostic Factors

Variables	B	*P*	*HR* (95%CI)
Pre-PLR (Low/ High)	0.581	0.003	1.789(1.216-2.631)
Pre-MLR (Low/ High)	0.686	0.001	1.986(1.353-2.914)
Post-PLR (Decrease/Rise)	0.348	0.068	1.417(0.975-2.058)
Post-NLR (Decrease/Rise)	0.310	0.106	1.363(0.936-1.985)
Age	0.002	0.860	1.002(0.984-1.020)
Previous treatment (S*/ S & C*/ C*)			
S & C	1.047	0.014	2.850(1.235-6.576)
C	0.939	0.029	2.557(1.100-5.942)

The overall Omnibus test of the model was significant (P < 0.001). S*: Surgery; S & C*: Surgery combined with chemoradiotherapy; C*: Chemoradiotherapy.

### Model validation

**Figure 3:**
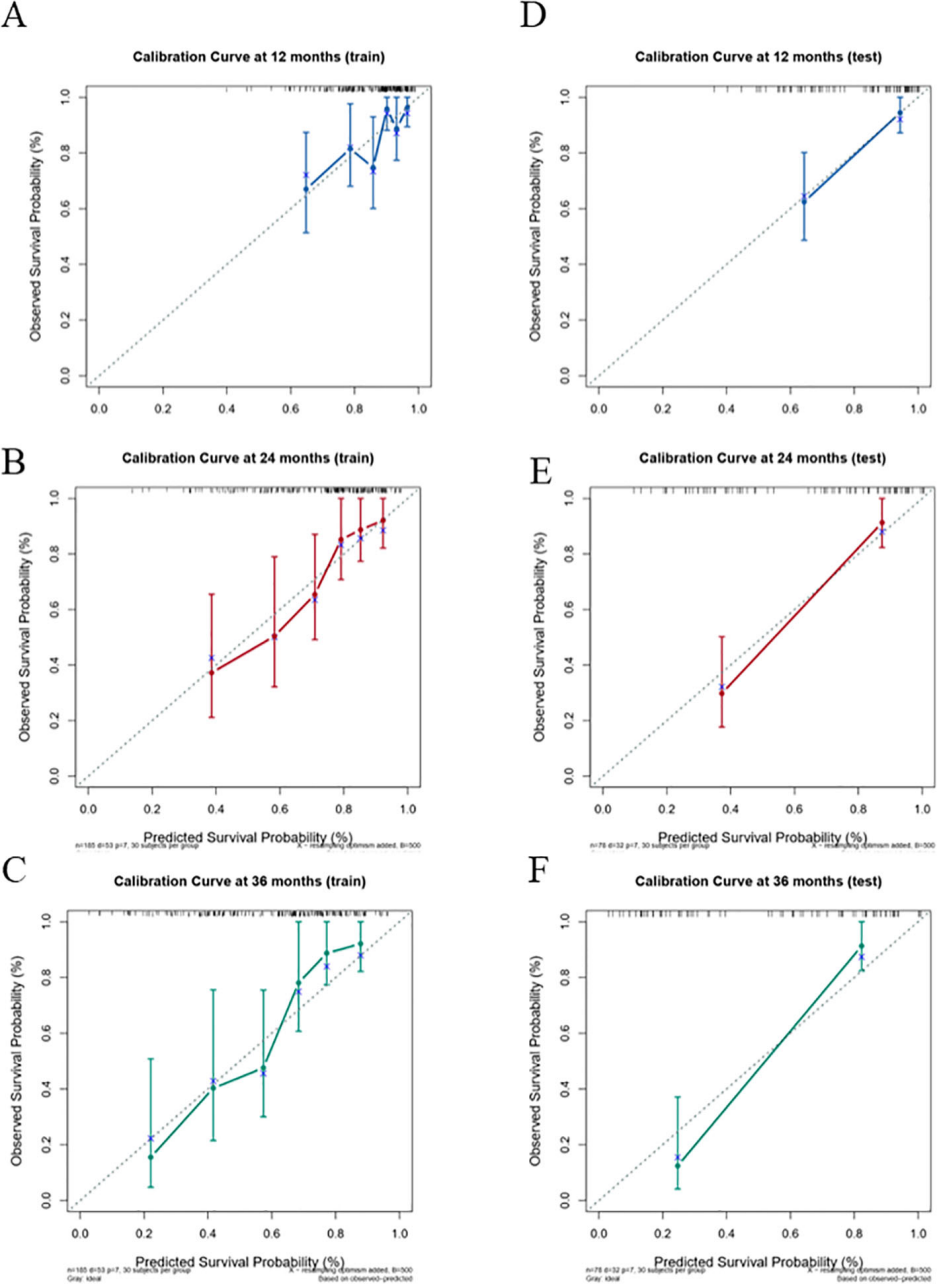
Calibration curves of the least absolute shrinkage and selection operator-Cox model for predicting 1-, 2-, and 3-year overall survival after immunotherapy. **(A–C)** Training set; **(D–F)** test set.

**Figure 4:**
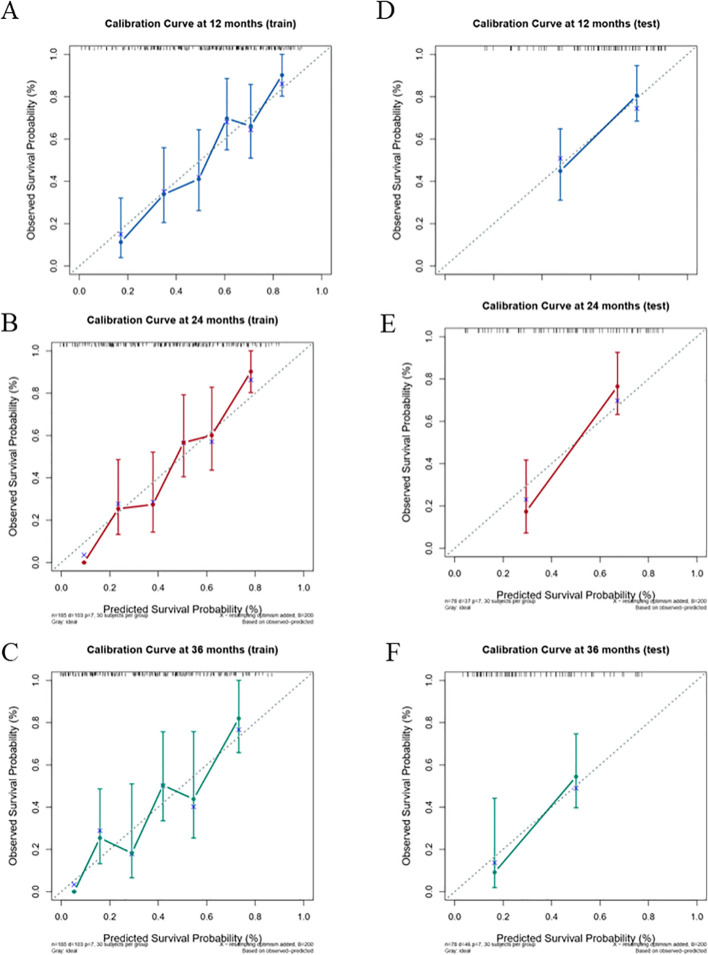
Calibration curves of the least absolute shrinkage and selection operator-Cox model for predicting 1-, 2-, and 3-year progression-free survival after immunotherapy. **(A–C)** Training set; **(D–F)** validation set.

**Figure 5:**
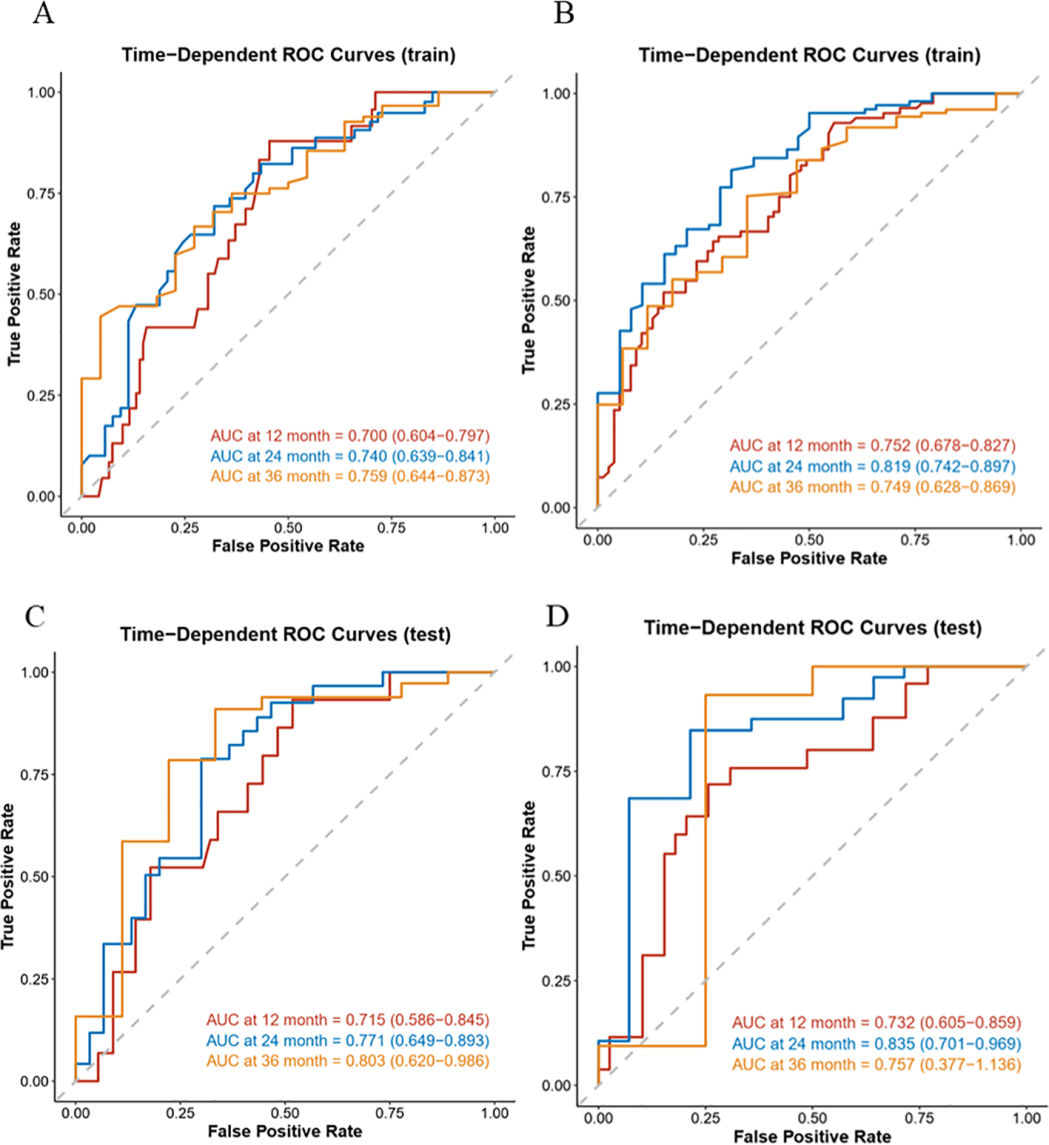
Receiver operating characteristic curves for evaluating the predictive performance of the least absolute shrinkage and selection operator-Cox model in the training and test sets. **(A)** Training set: values of area under the receiver operating characteristic curve (AUC) for predicting 1-, 2-, and 3-year overall survival were 0.700 (95% CI: 0.604–0.797), 0.740 (95% CI: 0.639–0.841), and 0.759 (95% CI: 0.644–0.837), respectively; **(B)** training set: corresponding AUC values for progression-free survival prediction were 0.752 (95% CI: 0.678–0.827), 0.819 (95% CI: 0.742–0.897), and 0.749 (95% CI: 0.628–0.869), respectively. **(C)** Test set: receiver operating characteristic curve analysis confirmed satisfactory predictive efficacy for overall survival, with AUC values of 0.715 (95% CI: 0.586–0.845), 0.771 (95% CI: 0.649–0.893), and 0.803 (95% CI: 0.620–0.986) for 1-, 2-, and 3-year endpoints, respectively; **(D)** test set: corresponding AUC values for progression-free survival prediction were 0.732 (95% CI: 0.605–0.859), 0.835 (95% CI: 0.701–0.969), and 0.757 (95% CI: 0.377–1.136), respectively.

**Figure 6:**
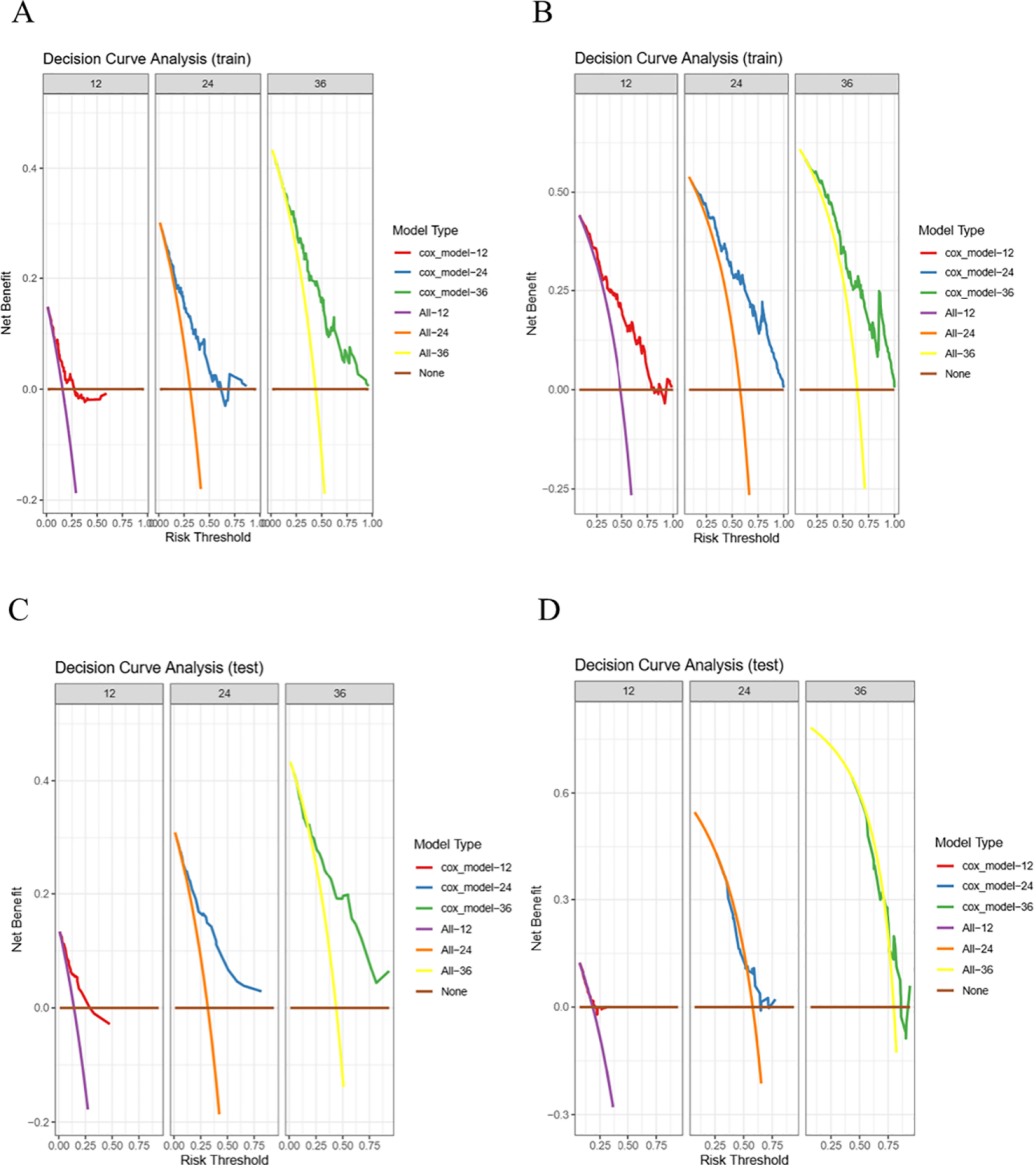
Decision curve analysis of the clinical utility of the least absolute shrinkage and selection operator-Cox model in the training and test sets. **(A, B)** Overall survival and progression-free survival in the training set: decision curve analysis confirmed that the model conferred greater net benefits than the two extreme intervention strategies for clinical decision-making. **(C, D)** Overall survival and progression-free survival in the test set: decision curve analysis confirmed that the model conferred greater net benefits than the two extreme intervention strategies for clinical decision-making.

**Figure 7:**
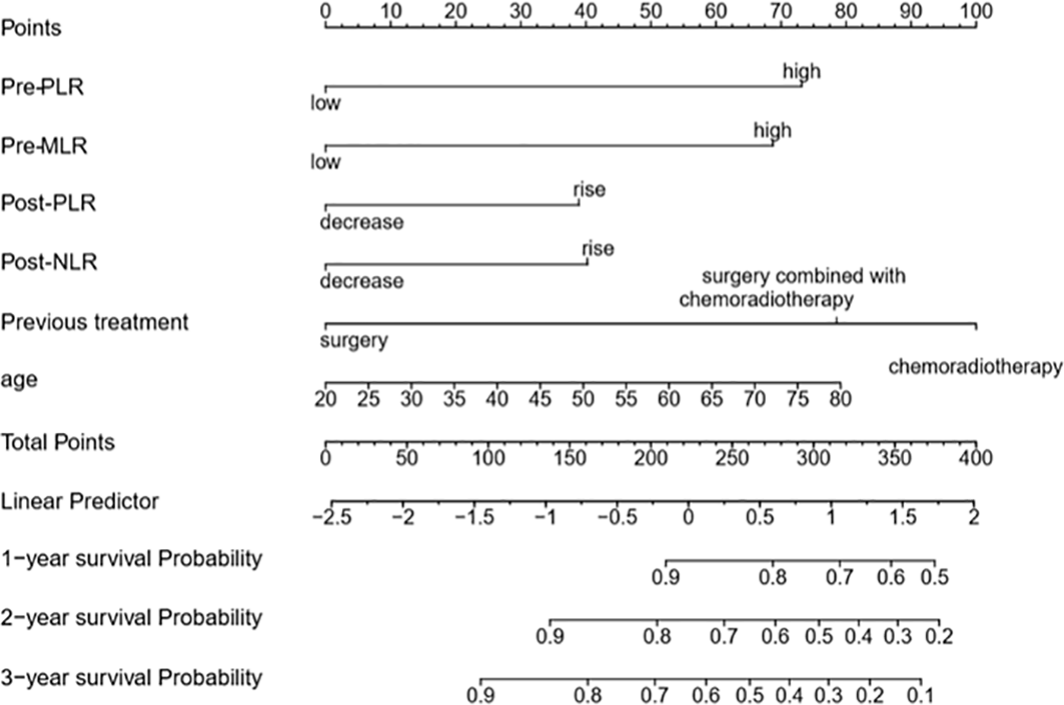
Nomogram for predicting the efficacy of PD-1/PD-L1 inhibitors in recurrent or metastatic cervical cancer.

**Figure 8:**
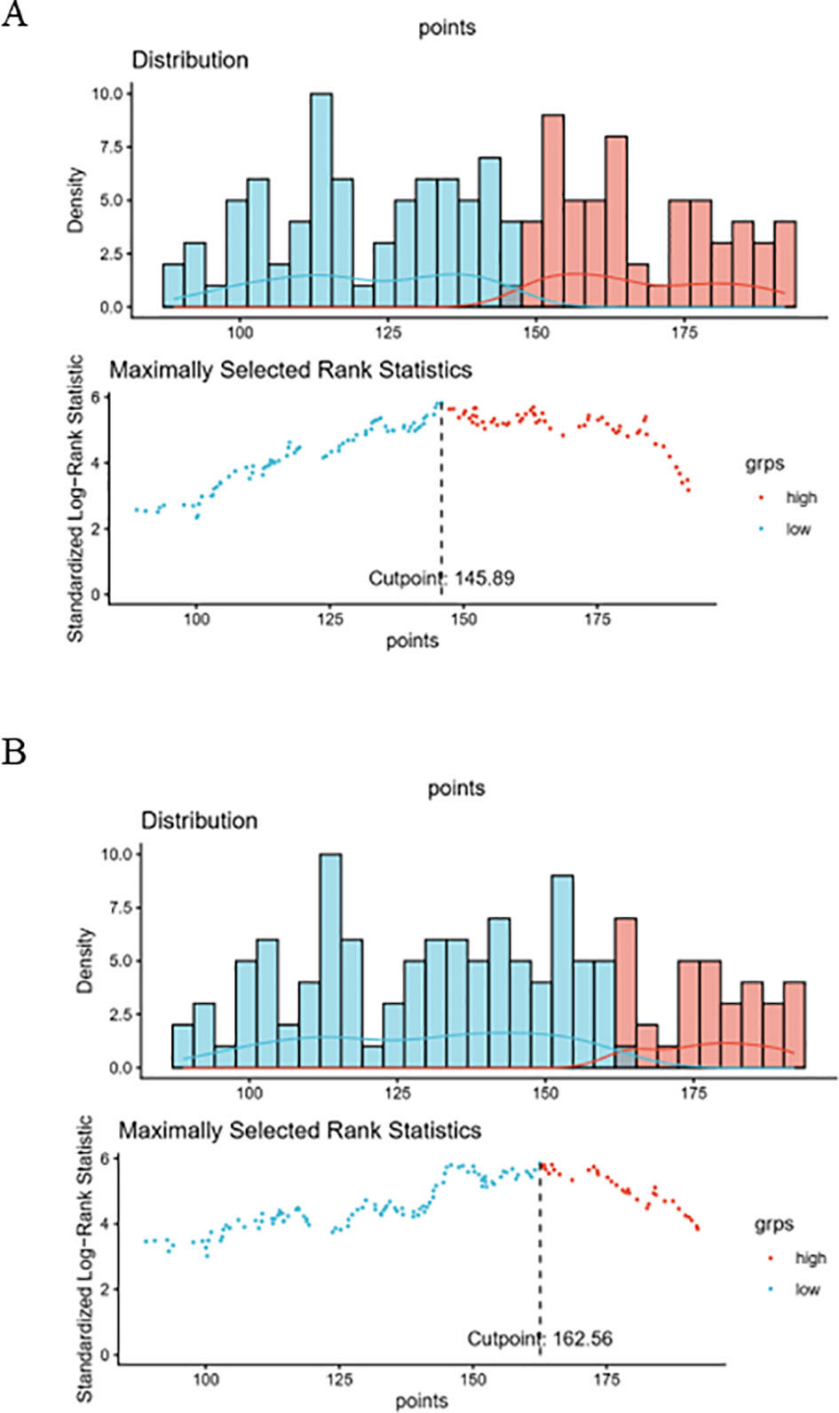
Screening of cutoff values for OS/PFS risk stratification by the maximally selected log−rank test. **(A)** Cutoff value for OS: 145.89. **(B)** Cutoff value for PFS: 162.56.

**Figure 9:**
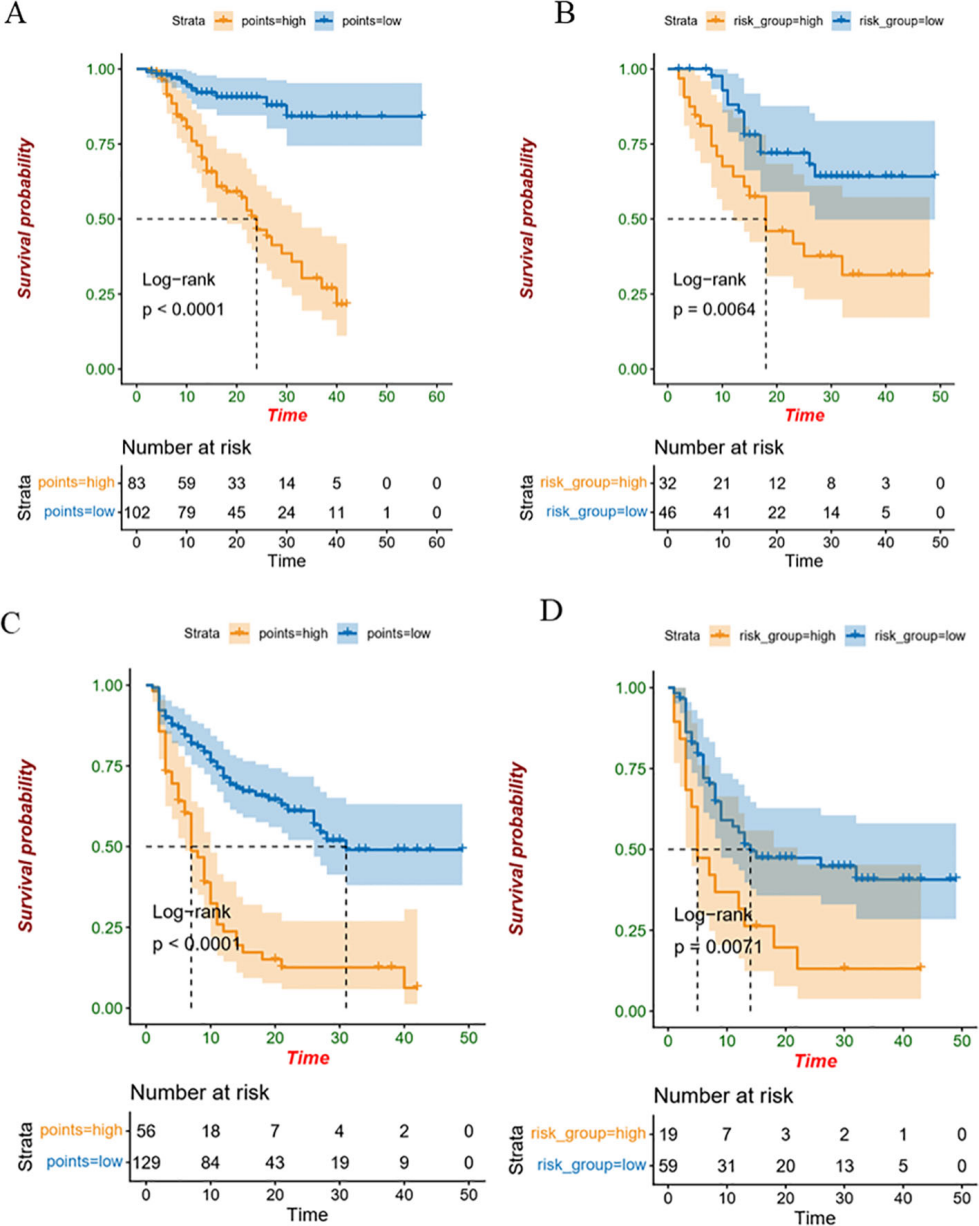
Kaplan–Meier survival curves for evaluating nomogram-based risk stratification in the training and test sets. **(A)** Overall survival of the training set: high-risk vs. low-risk group (P < 0.0001). **(B)** Overall survival of the test set: high-risk vs. low-risk group (P = 0.0064). **(C)** Progression-free survival of the training set: high-risk vs. low-risk group (P < 0.0001). **(D)** Progression-free survival of the test set: high-risk vs. low-risk group (P = 0.0071).

Calibration curves demonstrated favorable consistency between the predicted and actual observed outcomes in both the training set and the validation set, and this consistency was confirmed for both OS and PFS (**Figures 3**, **4**). To evaluate the predictive performance of the model, we first calculated the concordance index (C-index). In the training set, the C-index for OS was 0.704 (95% CI: 0.612–0.796), and that for PFS prediction was 0.692 (95% CI: 0.611–0.773). In the validation set, the C-indices for OS and PFS prediction increased to 0.763 (95% CI: 0.645–0.881) and 0.731 (95% CI: 0.622–0.840), respectively, indicating that the model had discriminatory ability for patient survival stratification. Subsequently, time-dependent ROC curve analysis was performed to further assess the predictive efficacy of the model. In the training set, the area under the ROC curve (AUC) values for 1-, 2-, and 3-year OS prediction were 0.700 (95% CI: 0.604–0.797), 0.740 (95% CI: 0.639–0.841), and 0.759 (95% CI: 0.644–0.837), respectively (**Figure 5A**). For PFS, the corresponding AUC values were 0.752 (95% CI: 0.678–0.827), 0.819 (95% CI: 0.742–0.897), and 0.749 (95% CI: 0.628–0.869), respectively (**Figure 5B**). Consistently, the validation set also showed good predictive performance for survival outcomes. The AUC values for 1-, 2-, and 3-year OS were 0.715 (95% CI: 0.586–0.845), 0.771 (95% CI: 0.649–0.893), and 0.803 (95% CI: 0.620–0.986), respectively (**Figure 5C**). For PFS, the corresponding AUC values were 0.732 (95% CI: 0.605–0.859), 0.835 (95% CI: 0.701–0.969), and 0.757 (95% CI: 0.377–1.136), respectively (**Figure 5D**). Collectively, these indicators present complementary predictive advantages, thereby improving the overall performance of the model. The DCA results showed that the model provided greater net benefits than the two extreme intervention strategies for clinical decision-making. This clinical value was stable in both the training (**Figures 6A, B**) and validation (**Figures 6C, D**) sets for both OS and PFS outcomes. Finally, the complex mathematical model developed in this study was converted into a nomogram (**Figure 7**). After summing the scores corresponding to each variable in the cumulative model, the cutoff value was determined using the maximally selected rank statistics, which divided patients into high-risk and low-risk groups. The cutoff value for predicting OS was 145.89 (**Figure 8A**), and the cutoff value for predicting PFS was 162.56 (**Figure 8B**). Kaplan–Meier survival analysis was performed to assess the prognostic value of nomogram-based risk stratification in both the training and validation sets. The results revealed consistent trends across the two cohorts: patients in the high-risk group exhibited significantly worse OS (training set, P < 0.0001; validation set, P = 0.0064) and PFS than those in the low-risk group (training set, P < 0.0001; validation set, P = 0.0071) (**Figure 9**).

## Discussion

Peripheral blood, the most commonly used sample for evaluating systemic inflammation, contains core inflammatory cells (20), which regulate the tumor microenvironment and antitumor immunity, supporting the screening of efficacy-predictive biomarkers. In cervical cancer, studies over the past decade have confirmed inflammation-related indicators as effective prognostic markers (21–23). For example, Trinh et al. found NLR levels (pre-, during, post-treatment) correlated with PFS and OS in patients receiving palliative chemotherapy, while Chen et al. showed high pre-treatment NLR (>2.4) shortened PFS (HR: 3.15, P = 0.002) and OS (HR: 3.83, P = 0.015) in those undergoing radical radiotherapy (24, 25). In immunotherapy, Calo et al. (49 patients) and Cheng et al. (70 patients) confirmed that low pre-treatment NLR (cutoffs 8 and 5.33, respectively) predicted better prognosis in r/m CC patients (26, 27), but these small-sample retrospective studies lacked dynamic indicator monitoring and multi-marker analysis. Liu et al. recently constructed PFS/OS predictive nomograms for r/m CC immunotherapy (204 patients) using LASSO and Cox analysis (C-indices 0.706/0.769, 1–2-year AUC up to 0.880), validated by TCGA/GEO datasets (28), highlighting the value of integrating inflammatory, tumor, and nutritional markers—consistent with Yan et al.’s nomogram (AUC = 0.897) (29). Notably, some variables insignificant in univariate analysis became key predictors in multivariate models, emphasizing the value of multi-factor integration. The predictive value of a single variable may be obscured by individual differences and confounding factors, but its combination with other variables can create a “combinatorial predictive advantage” through complementary effects, providing important methodological implications for similar research settings.

Based on the above research background and current status, this study enrolled 263 patients with r/m CC who received immunotherapy and had complete serological data. LASSO-Cox regression analysis was used to construct a clinical model. Our study showed that patients in the low pre-PLR group, low pre-MLR group, post-MLR decrease group, and post-NLR decrease group exhibited significantly improved OS and PFS, further verifying the stability and reliability of circulating inflammatory markers for prognostic assessment in patients with CC. It should be noted that although some variables included in the multivariate Cox model did not reach statistical significance (P > 0.05), they were retained after LASSO regression screening because of their complementary and synergistic predictive value with other variables. LASSO regression selects variables based on combined predictive performance rather than individual significance. Some variables may not show independent prognostic value alone, but they contribute to a more comprehensive risk assessment when integrated with others. This phenomenon reflects the multifactorial nature of tumor prognosis, where the joint effect of multiple factors is stronger than any single marker. The final model achieved satisfactory predictive efficacy, confirming that the inclusion of these variables improved the overall predictive power rather than reducing model stability.

### Effect of inflammatory markers on immunotherapy prognosis

How inflammatory markers affect prognosis in patients with cancer is unclear. However, studies indicate the involvement of regulatory pathways from the perspective of cellular function. Platelets promote tumor progression by enhancing angiogenesis and adhesion molecule production (30, 31). Neutrophilia is regulated by cytokines, such as IL-1β, IL-6, G-CSF, and VEGF, secreted by stromal and tumor cells (32, 33). Neutrophils can be classified into distinct subsets based on centrifugal density: normal or high-density neutrophils exert antitumor activity, whereas low-density neutrophils exert pro−tumor and immunosuppressive functions in the tumor microenvironment, and can be transformed from high−density neutrophils under microenvironmental regulation (34). Tumor−associated neutrophils drive tumor proliferation and metastasis via neutrophil elastase and MMP−9 (35–37), and suppress T−cell antitumor immunity through PD−L1/PD−1 interactions (36, 38). As key antitumor effector cells, lymphocytes directly determine therapeutic efficacy. Activated CD8+ T cells mediate tumor lysis via perforin and granzyme (39), while CD4+ helper T cells enhance antitumor immune responses by secreting IL-2, TNF-α, and IFN-γ (40). γδ T cells exert potent cytotoxicity and IFN−γ secretion (41, 42), and their high infiltration in cervical cancer is associated with a favorable prognosis (43). Peripheral blood monocytes, as precursors of key immune cells in the tumor microenvironment, also affect treatment outcomes (44). They differentiate into tumor−associated macrophages, dendritic cells and myeloid−derived suppressor cells to modulate tumor immunity (45, 46). Tumor−associated macrophages, the major monocyte derivative, promote tumor recurrence by suppressing T−cell immunity and sustaining angiogenesis (47). In multiple solid tumors, elevated peripheral blood monocyte counts correlate with poor immunotherapy response and shorter PFS, highlighting their value in predicting immunotherapy efficacy (48). These indicators provide complementary prognostic value, thereby improving the overall predictive performance of the model.

### Effect of age on immunotherapy prognosis

In this study, age was incorporated as a core predictive variable in the nomogram. Combined with the trends observed in clinical datasets, patients with r/m CC exhibited diminished immunotherapy efficacy with increasing age (49). Mechanistically, age-related immune senescence plays a central role. Aging causes thymic atrophy, reduces T-cell output and diversity, impairs CD8+/CD4+ T-cell function, and weakens tumor antigen recognition, all of which blunt antitumor immune responses elicited by immunotherapy (50). Elderly patients often have comorbidities that lower treatment tolerance, leading to dose modifications or discontinuation and further compromising efficacy. Additionally, aging-related chronic low-grade inflammation, together with impaired effector immune cell activity, aggravates tumor microenvironment immunosuppression and reduces immunotherapy benefit (51). Notably, Pawelec et al. reported an opposite finding in melanoma, where age did not affect immunotherapy predictive markers (52). This cancer-specific discrepancy may arise from differences in the biological characteristics of tumors: melanoma has a high tumor mutational burden, and elderly patients accumulate more genetic alterations and neoantigens. These neoantigens can be targeted by naive T cells, partly offsetting age-related immune decline and preserving immunotherapy efficacy (53).

### How do prior treatments affect prognosis?

The results of this study indicate that patients with different prior treatment histories exhibited significant prognostic differences following immunotherapy, although the underlying mechanisms remain unclear. We hypothesize that previous treatments may modulate the efficacy of immunotherapy by altering tumor burden, remodeling the tumor microenvironment, and regulating immune cell function. In clinical practice, early-stage CC patients (FIGO IB–IIA) usually undergo surgical resection (54), which achieves complete tumor removal, reduces tumor burden, and decreases the release of immunosuppressive factors, leading to higher sensitivity to immunotherapy at recurrence (55). By contrast, patients receiving chemoradiotherapy often retain more residual tumor cells. Meanwhile, chemoradiotherapy extensively damages CD8+ and CD4+ T cells in peripheral blood and the tumor microenvironment, which aggravates lymphopenia and immune dysfunction, making subsequent immunotherapy less effective in reversing the immunosuppressive state.

In this study, we developed a prognostic model using six survival-related factors to predict immunotherapy efficacy in advanced CC. The model’s discrimination, calibration and clinical utility were assessed with internal validation. Several limitations exist. First, as a single-center retrospective study, selection bias was unavoidable. Determining optimal cutoffs within the same dataset also increased the overfitting risk. The cutoffs identified were thus cohort-specific and required external validation before clinical use. Second, the modest sample size and incomplete PD-L1 data restricted analyses of PD-L1–inflammatory marker interactions, limiting the model’s interpretability and clinical applicability. Third, heterogeneity in therapeutic effects may have been introduced by different anti–PD-1/PD-L1 agents and variable concurrent treatments. Subgroup or sensitivity analyses were not feasible owing to the real-world design. Future multi-center prospective studies with external validation will be performed to overcome these limitations.

## Data Availability

The raw data supporting the conclusions of this article will be made available by the authors, without undue reservation.
